# Epidemiology and Risk Factors for Nosocomial Infections in Left Ventricular Assist Device Recipients

**DOI:** 10.3390/life14020270

**Published:** 2024-02-17

**Authors:** Simone Mornese Pinna, Silvia Corcione, Elena Cavallone, Nour Shbaklo, Davide Vita, Ilaria De Benedetto, Giorgia Montrucchio, Daniela Pasero, Anna Chiara Trompeo, Andrea Costamagna, Luca Brazzi, Mauro Rinaldi, Massimo Boffini, Francesco Giuseppe De Rosa

**Affiliations:** 1 Department of Medical Sciences, Infectious Diseases, University of Turin, 10124 Turin, Italynour.shbaklo@edu.unito.it (N.S.); ilaria.debenedetto@edu.unito.it (I.D.B.); francescogiuseppe.derosa@unito.it (F.G.D.R.); 2 Tufts University School of Medicine, Boston, MA 02111, USA; 3 Department of Medical Sciences, University of Turin, 10124 Turin, Italy; elena.cavallone@edu.unito.it; 4 Department of Surgical Sciences, University of Turin, 10124 Turin, Italy; g.montrucchio@gmail.com (G.M.); andrea.costamagna@unito.it (A.C.); luca.brazzi@unito.it (L.B.); 5 Department of Anesthesia, Intensive Care and Emergency, Città della Salute e della Scienza University Hospital, 10126 Turin, Italy; atrompeo@cittadellasalute.to.it; 6 Department of Emergency, Anaesthesia and Intensive Care Unit, AOU Sassari, 07100 Sassari, Italy; dpasero@uniss.it; 7 Department of Surgical Sciences, Cardiac Surgery Division, University of Turin, 10124 Turin, Italymassimo.boffini@unito.it (M.B.)

**Keywords:** left ventricular assist device, LVAD, nosocomial infections, multidrug resistant, MDR

## Abstract

Left ventricular assist devices (LVADs) have been increasingly used as a valid option to improve the prognosis and reduce the symptoms of end-stage heart failure. However, long-term complications, mostly infections and coagulation disorders, are frequent. We described the epidemiology and risk factors for nosocomial infections (NIs) in a cohort of adult patients who underwent continuous-flow LVAD implant between January 2010 and December 2017 in Turin, Italy. Secondary outcomes were the prevalence of multidrug-resistant (MDR) bacteria and mortality. Results: Overall, 64 LVADs were implanted. A total of 32 (50%) patients experienced at least one episode of NI, with a total of 46 infectious events. VAD-related infections occurred in 22 patients (68.8%). Non VAD-related NIs occurred in 12 patients (37.5%), mainly low respiratory tract infections. Length of intensive care unit admission was a risk factor for NI (OR 1.224, 95%CI; 1.049, 1.429). Gram-negative bacilli were responsible for 58.8% of VAD-related infections and 79.5% of non-VAD related infections. In sixteen patients (50%), at least one episode of infection was related to an MDR strain. INTERMACS class and length of MV were independent risk factors for NIs by MDR strains (respectively, OR 2.12, 95%CI: 1.08, 6.80; *p* = 0.02 and OR 1.46, 95%CI: 1.07, 5.52, *p* = 0.047). In-hospital mortality was 6.3%. No differences in mortality were observed between infected and non-infected patients (*p* = 0.61) even when caused by MDR strains (*p* = 0.143). Conclusion: the rate of nosocomial infections in LVAD patients is associated with the length of ICU admission, and the etiology of nosocomial infection after LVAD implant is mainly due to GNB, including a high rate of MDR strains, especially KPC-KP and MDR PA.

## 1. Introduction

Heart failure (HF) is one of the leading causes of morbidity and disability worldwide. It affects over twenty million people worldwide [1]. The overall prevalence in the European adult population is 2%, reaching 10% among patients aged 70 years or over [2]. Despite optimal medical treatment, an increasing number of people are expected to develop advanced HF, resulting in one-year mortality of 25–75% [3]. In patients with end-stage heart failure, mechanical circulatory systems (MCS) or heart transplantation (HT) can be indicated [3]. While HT remains the gold standard of care, the shortage of donors and strict inclusion criteria limit its application [4]. Therefore, left ventricular assist devices (LVADs) have emerged as a valid alternative option, significantly improving the prognosis in patients with end-stage HF. LVADs restore cardiovascular output and help reduce symptoms of heart failure, and continuous-flow devices have replaced pulsatile-flow devices because of their improved reliability and longer duration [5,6]. According to recent INTERMACS and EUROMACS reports, LVAD use has increased over the last decade [7,8]. Indications for LVADs are bridge to transplant, bridge to candidacy in cases of reversible contraindications to HT, bridge to decision in cardiogenic shock until hemodynamic stabilization, bridge to recovery in acute settings or destination therapy for transplant-ineligible patients [3]. However, long-term outcomes are significantly constrained by complications, mostly infections and coagulation disorders, both in the perioperative setting and after discharge, increasing morbidity and hospital readmission [7,8,9]. Three categories of infection occur in patients supported by LVADs. A VAD-specific infection may involve any part of the cardiac device (pump, pocket, cannula, or driveline). VAD-related infections occur even in patients not supported by LVADs but are generally more common in the presence of the device, such as mediastinitis or infective endocarditis. Non-VAD infections are not related to the presence of the device (e.g., urinary tract infections) but are included as a category to provide a comprehensive description of infection in this unique population [6]. Previous studies of patients who received continuous-flow LVADs found that the rate of infection was 32 to 36.9 per 100 patient-years [10,11]. The most common infection associated with LVADs is driveline infection, and after the introduction of specific criteria for defining infections in this population, the reported rate of infection ranges from 16% to 25% [12,13]. The microorganisms responsible for VAD infections vary, but Gram-positive bacteria are the primary culprits. Coagulase-negative staphylococci, Staphylococcus aureus, are commonly isolated from patients with LVAD-specific infections because they are often present in the skin microbiota and are able to form biofilms on devices [14].

Among Gram-negatives, Pseudomonas aeruginosa and Enterobacterales account for less frequent but severe infections. Yeast infections are not common in this setting [10].

Previous studies evaluating risk factors in the LVAD population involved pulsatile devices, while continuous devices are predominantly used now. These studies showed the effects of duration of LVAD support, renal failure, and higher body mass index on the risk of infection [10,15,16].

This study aims to describe the epidemiology of and risk factors for the development of nosocomial infections occurring during a 2-year follow up period in a cohort of patients who underwent continuous-flow LVAD implant in a primary and secondary referral cardiosurgical unit in Turin, Italy.

## 2. Materials and Methods

In this monocentric retrospective analysis, we reviewed all patients ≥18 years of age with a diagnosis of end-stage heart failure who received a continuous-flow LVAD implant between January 2010 and December 2017 at A.O.U. City of Health and Sciences, Turin. During the study period, different models of devices were implanted at our institution: Berlin Heart INCOR system (Berlin Heart AG, Berlin, Germany), Jarvik 2000 (Jarvik Heart, Inc., New York, NY, USA), Berlin Heart EXCOR (Berlin Heart Mediprodukt GmbH, Berlin, Germany), the HVAD^®^ (HeartWare^®^, Medtronic, Dublin Ireland), HeartMate II (Thermo Cardiosystems, Inc, Woburn, MA, USA), and the HeartMate III (Abbott, Abbott Park, IL, USA). Demographic, clinical, and microbiological data were collected retrospectively through reviewing medical charts or outpatient records of outpatient visits. Due to the non-interventional nature of this study, informed consent was not required for data collection. Patients who died within 48 h from VAD implantation were excluded. Risk factors were analyzed as follows: patient-related risk factors: underlying comorbidities, etiology of cardiac failure, INTERMACS scale, and indication for LVAD implant; procedure-related: procedure duration, length of extracorporeal circulation, mechanical ventilation and intensive care unit (ICU) stay, intra-aortic balloon pump (IABP), or ECMO (extracorporeal membrane oxygenation) requirement before or during LVAD implant. The primary outcome of the study was to investigate risk factors for nosocomial infections and the secondary outcomes were the prevalence of multidrug-resistant (MDR) bacteria and their impact on the in-hospital mortality during a 2-year follow-up period.

### 2.1. Definitions

In 2017, a consensus from the International Society for Heart and Lung Transplantation (ISHLT) revised the diagnostic criteria for infections in patients supported by VADs [8]. The ISHLT defined three categories of infections occurring during VAD support: VAD-specific infections can affect any part of the device (pump, cannula, pocket, or driveline); VAD-related infections may also occur in people not supported by cardiac assist devices but are more common in VAD recipients; non-VAD-related infections are not related to the presence of the device [7]. Nosocomial infections were defined according to the Centers for Disease Control and Prevention (CDC) definition [9].

For the purpose of this study, only infections occurring during hospitalization and after 24 h from implantation to 72 h after hospital discharge or LVAD removal were considered.

### 2.2. Statistical Analysis

Descriptive analysis was used to analyze the clinical characteristics of the patients included in this study. Categorical variables and continuous variables were studied by univariate analysis through logistic regression models. We analyzed the incidence of all types of infections (VAD-specific, VAD non-specific, and VAD-related occurring during hospitalization in patients who underwent LVAD implant). In the multivariable analyses, categorical covariates with missing information were imputed by the most frequent class or through the median of the available values (continuous covariates). A *p* value less than 0.05 was considered significant. Statistical analyses were performed using SPSS 23.0 (released 2015; IBM SPSS Statistics, Version 23.0. IBM Corp., Armonk, NY, USA). Discrete variables were expressed as percentages, whereas continuous variables were reported as medians with a 25th to 75th interquartile range (IQR).

### 2.3. Microbiological Data Collection

Identification of microorganisms and determination of antimicrobial susceptibility profiles were conducted with the Microscan Walkaway 96 plus system (Beckman Coulter, Brea, CA, USA), according to EUCAST criteria. Intravenous cefazoline or vancomycin, in cases of penicillin allergy, were adopted as surgical prophylaxis at implantation. Isolates were classified according to the criteria of Magiorakos et al. as multidrug-resistant or extensively drug-resistant [17].

Patients nasal swabs were screened pre-operatively to assess *S. aureus* carriage according to clinical practice. In cases of nasal carriage, a combination of 2% chlorhexidine bathing and nasal mupirocin was used.

### 2.4. Antibiotic Prophylaxis

Antibiotic prophylaxis was administered within 60 min prior to performing surgery with cefazolin at 2 g iv. In cases of β-lactam allergy, clindamycin at 600 mg was instead administered. In patients who were carriers of oxacillin-resistant *S. aureus*, a weight-adjusted dose of 15–20 mg/kg iv was administered. 

## 3. Results

During the study period, 64 LVADs were implanted in 64 patients. The baseline characteristics of our population at the time of implant are shown in Table 1. Patients were predominantly males (*n* = 54; 84%), with a median age at the time of implant of 61 years (IQR 56–66 years). The most frequent comorbidities were arterial hypertension (40%), dyslipidemia (28%), chronic kidney disease (28%), and diabetes in 17%. The majority of patients had an INTERMACS class <4 (*n* = 54; 64%). The etiology of heart failure was dilatative cardiomyopathy in 31 patients (48.4%), ischemic cardiomyopathy in 32 patients (50%), and hypertrophic cardiomyopathy in 1 patient. LVADs were implanted as destination therapy in 25 patients (39.1%), while in 22 patients (34.4%), the indication was bridge to transplant. All implanted devices were continuous flow; the most common one was Heartware HVAD (*n* = 45, 70%) and less frequently, Hearthmate-II and Jarvik 2000. The median length of hospital stay (LOS) was 43 ± 22.35 days (range of 43.6–119), including days before implantation. The length of surgery was 242 ± 69.44 min (range of 165–460 min), the average length of extracorporeal circulation (CEC) was 76 ± 58 min (range of 30–227 min), and the median time of ICU (intensive care unit) stay was 4 ± 16.93 days (range of 1–69) after LVAD implant and were all calculated after the procedure was complete. The median length of mechanical ventilation after LVAD implant was 15 ± 49.94 h (range of 8.25–24). The median time of ICU stay was 4 days after LVAD implant. 

### 3.1. Nosocomial Infections

**Figure 1:**
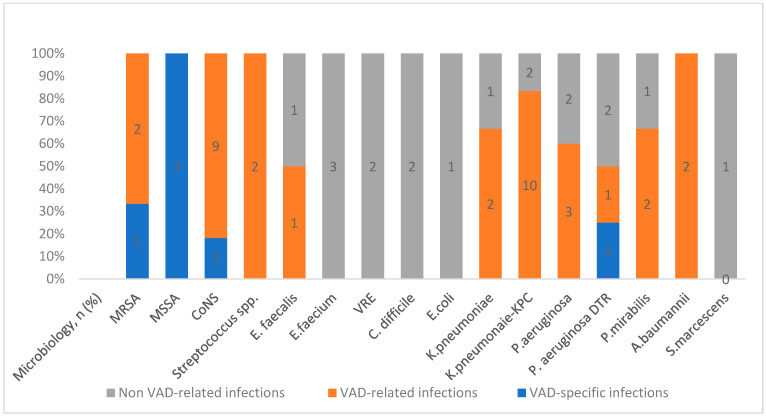
Microbiological isolates in nosocomial infections in LVAD patients. MRSA: meticillin-resistant *S. aureus*; MSSA: meticillin-susceptible *S. aureus*; CoNS: coagulase-negative *Staphylococci;* VRE: vancomycin-resistant *E. faecium*; KPC: *Klebsiella pneumoniae* carbapenemase; DTR: difficult-to-treat resistance.

A total of 32 (50%) patients experienced at least 1 episode of infection, with a total of 46 infectious events (16.5 infections per 1000 device days) (Figure 1). Seven patients experienced more than one episode of nosocomial infection during hospital admission. There were only three VAD-specific infections: two (6.3%, 0.7 infections per 1000 device days) surgical wound infections and one driveline infection, all requiring surgical debridement/source control. VAD-related infections were most frequent, occurring in 22 patients (68.8%), including 19 (6.8 infections per 1000 device days) bloodstream infections (BSI) and 6 (2.1 infections per 1000 device days) mediastinitis. In five patients, septic shock was present at the time of diagnosis. Non-VAD-related nosocomial infections were found in 12 patients (37.5%), 8 (2.9 infections per 1000 device-days) with low respiratory tract infections (LRTI, *n* = 8; 13%), followed by two C. *difficile* infections and two urinary tract infections (0.7 infections per 1000 device days; 3%).

### 3.2. Risk Factors for Nosocomial Infections 

Risk factors associated with the development of nosocomial infections were length of ICU stay (*p* < 0.0001), hospital length of stay (*p* < 0.001), and the necessity of continuous venovenous hemofiltration (*p* = 0.022). At the multivariate analysis level only, the length of ICU stay remained significant (OR 1.224, 95%CI; 1.049, 1.429) (Table 1). Overall, there were no significant differences between infected and non-infected patients in terms of baseline and pre-existing comorbidities.

### 3.3. Epidemiology of Nosocomial Infections

VAD-related infections were caused by Gram-negative bacilli (GNB) in 20 episodes (58.8%), while Gram-positive cocci, predominantly in polymicrobial infections, were isolated in 14 episodes (41.2%), mainly due to coagulase-negative staphylococci (CoNS). Interestingly, among GNB-isolated infections, 11 (64.71%) were caused by MDRo, mostly *K. pneumoniae* carbapenemase-producing strains (KPC-KP) and one MDR *P. aeruginosa*. Non-VAD-related nosocomial infections were polymicrobial in most of cases. Microorganisms causing non-VAD-related nosocomial infections were Gram-positive in eight cases (20.5%) cases. GNB were found in 10 patients (79.5%) including patients presenting with KPC-KP and MDR-*P. aeruginosa* infections.

### 3.4. Risk Factors for MDR Infections

Among 32 cases of infections, 16 patients experienced at least one episode due to an MDR strain. After splitting cases for MDR strains, we found that INTERMACS class (*p* = 0.016), length of ICU admission (*p* = 0.002), and length of MV (*p* = 0.036) were significantly associated with subsequent infection by MDR strains. INTERMACS class and length of MV remained significant even in the multivariate analysis (respectively, OR 2.12, 95%CI: 1.08, 6.80; *p* = 0.02 and OR 1.46, 95%CI: 1.07, 5.52, *p* = 0.047).

### 3.5. Outcome

**Figure 2:**
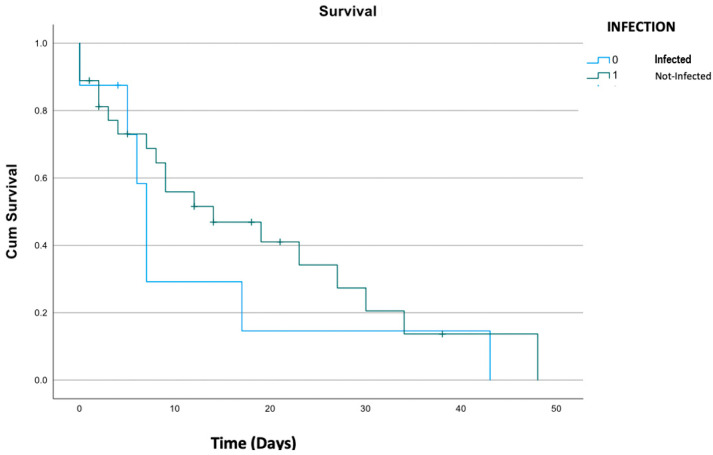
Kaplan–Meier survival analysis of patients with nosocomial infections compared to others.

Overall, in-hospital mortality was 6.3%, while 2-year mortality was 68.8. No differences in mortality were observed between patients with nosocomial infection and those without, as represented in Figure 2 (*p* = 0.61). As bloodstream infections and low respiratory tract infections are severe events and quite frequent in our population, we made a sub-group analysis on mortality among patients with BSI and nosocomial pneumonia. Mortality did not differ even in patients with BSI compared to others (respectively, *p* = 0.10 and *p* = 0.411).

Also, the mortality did not differ between infected and non-infected patients even when dividing the cases by type of infection or MDR characteristics (*p* = 0.143). 

## 4. Discussion

In this paper, we described the incidence of nosocomial VAD-related and non-LVAD-related infections in a series of 64 patients in a single center over an 8-year period, providing information on microbiological isolates including MDR strains. There are few data, mostly coming from single centers, available on the epidemiology of nosocomial infections in LVADs, a 2-year follow-up period. Nosocomial VAD-related and non-VAD-related infections are distributed early (<3 months) after VAD implant, compared to VAD-specific infections that occur late (>3 months). We decided to focus our study mostly on nosocomial infections different from LVAD-specific infections. MDRO infections are a serious complication, even life-threatening for LVAD recipients. In the recent literature, among pediatric patients supported by different VAD devices, 56% (infection rate of 17.6 per 1000 patient days) of patients developed at least one episode of healthcare-associated infections (HAI) [8]. Longer hospital and ICU admission stay were related to the development of HAI, as well as length of mechanical support. Interestingly, as many as 25% of isolates were MDR strains, mainly P. aeruginosa. The largest study on LVAD infections due to MDR organisms is that of Donahey et al., a single-center study including 57 patients with a lower number of nosocomial infections compared to our series (31% vs. 50%) but a higher prevalence of P. aeruginosa strains [18]. Similarly, other studies have detected a higher prevalence of P. aeruginosa, followed by Enterobacter spp. and Serratia spp., highlighting the importance of the local epidemiology [18,19]. Similar to previous reports, overall, BSI were common nosocomial LVAD-related events in our study (22 events, 34%). BSI were the most common type of LVAD-related infection overall and were significantly more prevalent compared to other infections. This suggests that these BSI are associated with risk factors such as frequent manipulation of central venous catheters or hematogenous spread from other distant sources such as bacteremia from infected indwelling urinary catheters, VAP, deep-seated wound infections or from cannulation sites in patients who required other forms of temporary cardiovascular support such as Impella or extracorporeal membrane oxygenation (ECMO). In fact, in our study, 58.8% of BSI were caused by GNB. As previously reported, the majority of these events could not be associated with the LVAD itself [20].

Among non-VAD-related events, lower respiratory tract infections were prevalent (13%). This finding is not surprising. Pneumonia was the most common type of non-LVAD-related infection, followed by C. difficile infections and urinary tract infections. Due to the severity of underlying disease and even the long ICU stay, nosocomial pneumonia is likely to occur within the first three months following implant. This suggests that many of these infections are hospital-acquired and occur while patients are still in the intensive care unit or during their hospital stay after implant. Nosocomial pneumonia requires further investigation to identify patient risk factors such as those with right heart failure, prolonged intubation, and bleeding disorders that could be associated with ventilator-associated pneumonia (VAP). The high rates of BSI and pneumonia are in line with the ISHLT Mechanically Assisted Circulatory Support Registry [21]. Nonetheless, infections associated with LVADs are a significant burden but do not necessarily preclude the transplantation even in patients with infections sustained by MDR strains. In fact, in our population, infection or colonization by an MDR strain is not *a priori* an exclusion criterion for transplantation. Our study is one of the most relevant in terms of MDR prevalence among people who underwent LVAD implant. In fact, among 46 infectious events (16.5 infections per 1000 LVAD days), GNB were the most common, accounting for 58.82% of patients. Interestingly, as many as 64.71% of GNB recovered from blood cultures were MDR strains [18,19,20,21,22]. The most common MDR organisms isolated in our study were KPC-KP followed by DTR-PA and VRE. Even non-VAD-related nosocomial infections were mainly related to GNB in 79.49% of patients, including 25.64% caused by KPC-KP and MDR-PA. Otherwise, the microbiology of LVAD-related infections in our center appears to be driven by KPC-KP and MDR-PA. This reflects the large prevalence of KPC-KP among Italian hospitals and its burden in critical environments such as cardiovascular surgery [23,24]. Furthermore, patients who developed MDR-related infections had a significantly longer time of MV and a worse INTERMACS class compared to others. This finding has been reported previously in the literature, confirming the relevance of the severity of baseline conditions and ecological hospital pressure as main factors for the development of MDRo infections in patients supported by different MCS [24]. In our series, INTERMACS (*p* = 0.016, OR 2.12, 95%CI: 1.08, 6.80) class and length of MV (*p* = 0.02 and OR 1.46, 95%CI: 1.07, 5.52, *p* = 0.047) were identified as significantly associated with the development of nosocomial infections due to MDR strains. Interestingly, LRTI followed by bloodstream infections were the most common nosocomial infection during hospitalization. The mean ICU stay was significantly associated with the development of nosocomial infections (*p* < 0.0001, OR 1.224, 95%CI (1.049–1.429). However, the infections did not contribute significantly to mortality (*p* = 0.61) even when analyzing the sub-group infected with MDR strains (*p* = 0.143). Nonetheless, in this latter sub-group, we observed a trend of higher mortality (18.2%) compared to patients infected with non-MDR strains (4.8%). In larger studies, there was a clear impact of MDR organisms, including KPC-KP, on mortality [25,26]. One possible explanation is that the recent implementation of infection surveillance programs may reduce the time to effective target therapy among patients carrying MDR strains. Furthermore, among patients colonized by MDR strains, initial antimicrobial use was extensive and frequently based on combinations of antibiotics with antimicrobial activity against the strain, maximizing the chances of using at least one antibiotic active against the strains, although we did not assess the appropriateness of antimicrobial prescription. Furthermore, MDR strains seem more common among patients with a prolonged length of MV and worse INTERMACS class compared to patients infected with non-MDR strains; however, further studies are warranted to confirm this hypothesis. This result would be expected to be related to the higher co-morbidity and severity of underlying cardiac diseases among patients with a low INTERMACS score. In the study by Zhou et al., mortality was significantly higher in patients with post-operative infections regardless of the number of infectious events, and patients with infections less frequently were candidates for heart transplant [27]. Interestingly, we noted three cases of early exit-site or driveline infections in the early post-implant period. These cases were sustained by Gram-positive cocci. These early events may be directly related to the implant surgery and documented as soft tissue infections or can be acquired in the hospital after implant during manipulation of the driveline and exit site, mainly during change in medication [28]. The challenges of nosocomial infections in patients supported by LVADs is related to the lack of robust evidence for key aspects due to the presence of a VAD, which complicates the clinical diagnosis, treatment, and source control. Furthermore, when facing chronic specific LVAD infections, weighing the chance of microbial cure against the risk of side effects and consequences of breakthrough infection is tiresome and treatment options are limited. Hence, this particular field can facilitate opportunities for improved antimicrobial stewardship and improved patient care through improved outpatient parenteral antimicrobial therapy (OPAT) programs and a multidisciplinary management team with expertise in LVAD management [29]. Infection control programs must be equipped with the necessary resources to conduct a thorough surveillance of healthcare-associated infections, including surgical site infections, VAP, central venous line-related infections, catheter-related urinary tract infections, antimicrobial prophylaxis, dressings, and management of the exit site and drivelines infections, with detailed microbiology and susceptibility testing in this vulnerable population [30,31]. A substantial portion of these infections can be prevented by implementing well-documented and successful healthcare bundles and multimodal interventions from previously published studies in intensive care units [32]. Substantial opportunities exist to enhance outcomes by reducing healthcare-associated infections during the first three months, which will have a positive and significant impact on long-term survival rates for patients supported by LVADs. Thus, improvement in antimicrobial prescriptions through tailored interventions based on microorganism-isolated susceptibility patterns is needed in order to reduce treatment-related adverse events, particularly in patients with destination therapy. Furthermore, these data highlight the relevance of surveillance protocols to monitor indwelling devices at risk of nosocomial infections such as central venous lines, urinary catheters, and endotracheal tubes. This study has several limitations. Although this study included a considerable number of patients with nosocomial infections, including MDR organisms, the small sample size may have reduced the statistical power and the capacity to draw conclusions. Nevertheless, the comparison of infected and non-infected patients, the duration of follow-up, and the focus on MDR strains may be worth reporting. Second, the retrospective design of this study will always be limited by bias and confounding, which, in our case, is limited by the stability of multidisciplinary teams treating patients.

## 5. Conclusions

In conclusion, the rate of nosocomial infections in LVADs is associated with the length of ICU admission, and the etiology of nosocomial infection after LVAD implant is mainly due to GNB, including a high rate of MDR strains, especially KPC-KP and MDR PA.

## Figures and Tables

**Table 1:** Overall population analysis of baseline characteristics.

Baseline Characteristics	Overall	Nosocomial Infection (*n* = 32)	No Infection (*n* = 32)	*p* Value	Multivariate Analysis (95%CI)
Age (years, IQR)	61 (56–65)	62.5 (61.5–65)	62.5 (61.5–65)	0.458	
Males (%)	54 (84%)	27 (84.4%)	27 (84.4%)	0.340	
Hypertension (%)	26 (40%)	12 (37.5%)	14 (43.8%)	0.728	
Diabetes (%)	11 (17%)	4 (12.5%)	7 (21.9%)	0.356	
Dyslipidemia (%)	18 (28%)	7 (21.9%)	11 (34.4%)	0.448	
COPD (%)	5 (6%)	3 (9.4%)	2 (6.3%)	0.999	
Chronic kidney disease (%)	18 (28%)	8 (25.0%)	10 (31.3%)	0.999	
INTERMACS Level (%)					
1	7 (10.9%)	5 (15.6%)	2 (6.3%)		
2	13 (20.3%)	9 (28.1%)	4 (12.5%)		
3	34 (53.1%)	14 (43.8%)	20 (62.5%)	0.259	
4	8 (12.5%)	2 (6.3%)	6 (18.8%)		
5	1(1.6%)	1 (3.1%)	0 (0%)		
6	1 (1.6%)	1 (3.1%)	0 (0%)		
Indication for LVAD (%)					
BTT	22 (34.4%)	11 (34.0%)	11 (34.0%)		
BTC	17 (26.6%)	11 (34.0%)	6 (34.4%)	0.814	
DT	25 (39.1%)	10 (31.1%)	15 (46.9%)		
Type of LVAD support					
Hearthware	45 (70%)	27 (84.4%)	19 (59.4%)		
Heartmate II	17 (26.6%)	12 (37.5%)	5 (15.6%)	0.750	
Jarvic 2000	2 (3.1%)	0 (0%)	2 (6.3%)		
Cardiac disease (%)					
Dilated CM	31 (48.4%)	19 (59.4%)	12 (37.5%)	0.909	
Ischemic CM	32 (50.0%)	20 (62.5%)	12 (37.5%)		
Valvular	1 (1.6%)	1 (3.1%)	0 (0%)		
Ventricular support pre-					
implant (%)					
IABP	22 (32.8%)	13 (40.6%)	9 (28.1%)	0.584	
ECMO	7 (10.9%)	6 (18.8%)	1 (3.1%)		
Other cardiovascular surgery during LVAD implant	5 (7.8%)	3 (9.4%)	2 (6.3%)	0.452	
Weight, (kg, IQR)	70 (60.0–79.5)	69.0 (60.0–80.0)	69.0 (60.0–75.5)	0.345	
Mean length of surgery (min, IQR)	242.5 (210.0–294.0)	257.5 (210.0–338.75)	235.0 (210.0–282.0)	0.080	
Mean ICU (days)	4 (3.0–11.5)	9.0 (3.0–24.75)	4.0 (2.0–5.0)	<0.0001	Sig. 0.022, OR 1.224; 1.049, 1.429
Mean time of mechanical ventilation (h)	18 (9.0–33.0)	23.0 (12.25–100)	11.0 (8.0–21.0)	0.070	Sig. 0.622, OR 0.99; 0.973, 1.013
Mean length of hospital stay (days)	37.5 (28–56)	50.5 (34.0–61.75)	31.0 (23.75–45.75)	<0.001	Sig. 0.119, OR 1.031; 0.992, 1.070
Mean time of ECC (min)	76 (59.3–105.3)	79.0 (60.0–109.0)	66 (55.0–101.5)	0.272	Sig. 0.470, OR 0.99; 0.962, 1.018
CVVH (%)	12 (18.8%)	10 (31.3%)	2 (6.3%)	0.022	Sig. 0.879, OR 0.88; 0.194, 4.069
In-hospital mortality, n (%)	4 (6.25%)	3 (9.4%)	1 (3.1%)	0.613	

COPD: chronic obstructive pulmonary disease; BTT: bridge to transplant; BTC: bridge to candidacy; DT: destination therapy; CM: cardiomyopathy; IABP: intra-aortic balloon pump; ECMO: extracorporeal membrane oxygenation; ECC: extracorporeal circulation; ICU: intensive care unit; LVAD: left ventricular assist device.

## Data Availability

The data that support the findings of this study are available from the corresponding author, [SMP], upon reasonable request.
